# Motor Preparation Disrupts Proactive Control in the Stop Signal Task

**DOI:** 10.3389/fnhum.2018.00151

**Published:** 2018-05-04

**Authors:** Wuyi Wang, Sien Hu, Jaime S. Ide, Simon Zhornitsky, Sheng Zhang, Angela J. Yu, Chiang-shan R. Li

**Affiliations:** ^1^Department of Psychiatry, Yale University, New Haven, CT, United States; ^2^Department of Psychology, State University of New York, Oswego, NY, United States; ^3^Department of Cognitive Science, University of California, San Diego, La Jolla, CA, United States; ^4^Department of Neuroscience, Yale University, New Haven, CT, United States; ^5^Interdepartmental Neuroscience Program, Yale University, New Haven, CT, United States

**Keywords:** cognitive control, post-error slowing, post-signal slowing, motor urgency, motor readiness

## Abstract

In a study of the stop signal task (SST) we employed Bayesian modeling to compute the estimated likelihood of stop signal or P(Stop) trial by trial and identified regional processes of conflict anticipation and response slowing. A higher P(Stop) is associated with prolonged go trial reaction time (goRT)—a form of sequential effect—and reflects proactive control of motor response. However, some individuals do not demonstrate a sequential effect despite similar go and stop success (SS) rates. We posited that motor preparation may disrupt proactive control more in certain individuals than others. Specifically, the time interval between trial and go signal onset—the fore-period (FP)—varies across trials and a longer FP is associated with a higher level of motor preparation and shorter goRT. Greater motor preparatory activities may disrupt proactive control. To test this hypothesis, we compared brain activations and Granger causal connectivities of 81 adults who demonstrated a sequential effect (SEQ) and 35 who did not (nSEQ). SEQ and nSEQ did not differ in regional activations to conflict anticipation, motor preparation, goRT slowing or goRT speeding. In contrast, SEQ and nSEQ demonstrated different patterns of Granger causal connectivities. P(Stop) and FP activations shared reciprocal influence in SEQ but FP activities Granger caused P(Stop) activities unidirectionally in nSEQ, and FP activities Granger caused goRT speeding activities in nSEQ but not SEQ. These findings support the hypothesis that motor preparation disrupts proactive control in nSEQ and provide direct neural evidence for interactive go and stop processes.

## Introduction

Previously we combined computational modeling and fMRI of a stop signal task (SST) to characterize the neural processes linking conflict anticipation or Bayesian estimate of the likelihood of an upcoming stop signal—P(Stop)—and go trial reaction time (goRT; Ide et al., 2013; Hu et al., 2015a). A higher P(Stop) is associated with prolonged goRT, a behavioral finding related to “sequential effect” (Yu and Cohen, 2008). In brain imaging data, the anterior pre-supplementary motor area (preSMA) along with the inferior parietal cortex (IPC) respond to higher P(Stop) and the posterior preSMA and bilateral anterior insula (AI) respond to prolonged goRT. Granger causality analyses showed directional influence of anterior preSMA on posterior preSMA and bilateral insula, suggesting proactive control of motor response (Hu et al., 2015a). A sequential effect reflects trial by trial monitoring and learning to update the current expectation of the stop signal. On the other hand, some participants failed to demonstrate a significant sequential effect despite similar go and stop success (SS) rates. The current study aimed to examine the neural processes of proactive control that distinguish individuals who demonstrate the sequential effect (SEQ) and those who do not (nSEQ).

One hypothesis is that P(Stop) is not represented in nSEQ. That is, individuals may not track the occurrence of stop signal and thus do not demonstrate a sequential effect. As the true probability of the stop signal does not vary from trial to trial, tracking the stop signal does not confer advantages and nSEQ would not suffer in behavioral performance. An alternative hypothesis concerns motor preparation—a process that has not been systematically considered in characterizing SST performance. In the SST, the go signal appears after a randomized interval (1–5 s)—the fore-period (FP)—following trial onset. It is known that a longer FP is associated with motor preparation and correlated negatively with goRT, reflecting an urgency for action (Woodrow, 1914; Bertelson and Tisseyre, 1968; Niemi and Naatanen, 1981; Coull et al., 2016), or a “FP effect” (Li et al., 2005). The FP effect describes the readiness level at which participants are prepared to respond to the go signal. It is possible that individuals vary in this motor urgency, and preparatory motor activities in nSEQ play an outsized role in disrupting the neural processes of proactive control. Specifically, under this hypothesis, we expect P(Stop) to be represented in both SEQ and nSEQ, but SEQ and nSEQ may demonstrate distinct cerebral processes of motor preparation and proactive control. Alternatively, SEQ and nSEQ may share the same processes but demonstrate distinct regional interactions such that the sequential effect is disrupted in nSEQ.

We have three aims in this study. First, we characterized the FP effect and posited a stronger FP effect in the nSEQ than SEQ. Second, by modeling hemodynamic responses each at trial and go signal onsets we examined the neural correlates of conflict anticipation—expectation to encounter a stop signal—and goRT slowing as well as the correlates of motor preparation and goRT speeding. We posited that SEQ and nSEQ would demonstrate different patterns of regional activations. Third, with Granger causality analyses we examined Granger causal connectivities among regional activities of conflict anticipation, motor preparation, goRT slowing, and goRT speeding. We posited a stronger influence of motor preparation on conflict anticipation and goRT-related activities in nSEQ than SEQ. The study would address how control and action circuits determine a critical executive function and provide further evidence to support the Bayesian model of inhibitory control (Shenoy and Yu, 2011), where go and stop processes interact to support behavioral performance.

## Materials and Methods

### Participants and Behavioral Task

Healthy participants were recruited from the Greater New Haven area of Connecticut. All were without neurological or psychiatric illnesses, denied use of illicit substances and tested negative in urine toxicology on the day of study. A total of 116 subjects (32.3 ± 12.6 years of age; 59 men) participated in the study, following a protocol approved by the Human Subject Investigation Committee of Yale University School of Medicine. All subjects gave written informed consent in accordance with the Declaration of Helsinki.

Participants performed a SST (Li et al., 2009; Hu and Li, 2012), in which go and stop trials were randomly intermixed in presentation with an inter-trial-interval of 2 s. A fixation dot appeared on screen to signal the beginning of each trial. After a FP varying from 1 s to 5 s (uniform distribution), the dot became a circle—the “go” signal—prompting participants to quickly press a button. The circle disappeared at button press or after 1 s if the participant failed to respond. In approximately one quarter of trials, the circle was followed by a “cross”—the stop signal—prompting participants to withhold button press. The trial terminated at button press or after 1 s if the participant successfully inhibited the response. The time between the go and stop signals, the stop signal delay (SSD), started at 200 ms and varied from one stop trial to the next according to a staircase procedure, increasing and decreasing by 67 ms each after a successful and failed stop trial (Levitt, 1971). With the staircase procedure we anticipated that participants would succeed in withholding the response half of the time. Participants were trained briefly on the task before imaging to ensure that they understood the task. They were instructed to quickly press button when they saw the go signal while keeping in mind that a stop signal might come up occasionally. In the scanner, they completed four 10-min sessions of the task, with approximately 100 trials in each session.

### Behavioral Data Analysis

A critical SSD was computed for each participant that represents the time delay required for the participant to successfully withhold the response in half of the stop trials, following a maximum likelihood procedure (Wetheril et al., 1966). Briefly, SSDs across trials were grouped into runs, with each run being defined as a monotonically increasing or decreasing series. We derived a mid-run estimate by taking the median of every second run. The critical SSD was computed by taking the mean of all mid-run SSDs. It was reported that, except for experiments with a small number of trials (<30), the mid-run measure is close to the maximum likelihood estimate of X50 (50% positive response, Wetheril et al., 1966). The stop signal reaction time (SSRT) was computed for each participant by subtracting the critical SSD from the median goRT (Logan et al., 1984).

A sequential effect was quantified by Pearson correlation between P(Stop)—the Bayesian estimate of the probability of a stop signal—and RT of go trials for individual subjects. We used a dynamic Bayesian model (Yu and Cohen, 2008) to estimate the prior belief of an impending stop signal on each trial, based on prior stimulus history. In the model subjects believe that stop signal frequency *r_k_* on trial *k* has probability α of being the same as *r_k_* − 1, and probability (1-α) of being re-sampled from a prior distribution π(*r_k_*). Subjects also believe that trial *k* has probability *r_k_* of being a stop trial, and probability 1 − *r*_k_ of being a go trial. Based on these generative assumptions, subjects use Bayesian inference to update their prior belief of seeing a stop signal on trial *k*, *p*(*r_k_*|***s***_*k* − 1_) based on the prior on the last trial *p*(*r*_k − 1_|***s***_k − 1_) and last trial’s true category (*s*_k_ = 1 for stop trial, *s*_k_ = 0 for go trial), where ***s***_k_ = {*s*_1_, …, *s*_k_} is short-hand for trials 1 through *k*. Specifically, given that the posterior distribution was *p*(*r_k − 1_*|***s***_*k* − 1_) on trial *k − 1*, the prior distribution of stop signal in trial *k* is given by:

$$p(r_{k}|s_{k\;-\;1})\;=\;\alpha p(r_{k\;-\;1}|s_{k\;-\;1})\;+\;(1\;-\;\alpha )\pi (r_{k}),$$

where the prior distribution π(*r_k_*) is assumed to be a beta distribution with prior mean *pm*, and shape parameter *scale*, and the posterior distribution is computed from the prior distribution and the outcome according to the Bayes’ rule:

$$p\text{(}r_{k}\text{|}s_{k}\text{) }\propto \;P\text{(}s_{k}\text{|}r_{k}\text{)}p\text{(}r_{k}\text{|}s_{k}-1$$

The Bayesian estimate of the probability of trial *k* being stop trial, which we colloquially call P(Stop) in this article, given the predictive distribution *p*(*r_k_*|***s**_k − 1_*) is expressed by:

$$P\left(s_{k}=1|s_{k-1}\right)=\int P\left(s_{k}=1|r_{k}\right)P\left(r_{k}|s_{k-1}\right)dr_{k}=\int r_{k}P\left(r_{k}|s_{k-1}\right)dr_{k}=<r_{k}|s_{k-1}>$$

In other words, the probability P(Stop) of a trial *k* being a stop trial is simply the mean of the predictive distribution *p*(*r_k_*|***s***_*k* − 1_). The assumption that the predictive distribution is a mixture of the previous posterior distributions and a generic prior distribution is essentially equivalent to using a causal, exponential, linear filter to estimate the current rate of stop trials (Yu et al., 2009). In summary, for each subject, given a sequence of observed go/stop trials, and the three model parameters {α, *pm*, *scale*}, we estimated P(Stop) for each trial.

### Imaging Protocol and Spatial Preprocessing of Brain Images

Conventional T1-weighted spin-echo sagittal anatomical images were acquired for slice localization using a 3T scanner (Siemens Trio). Anatomical images of the functional slice locations were obtained with spin-echo imaging in the axial plan parallel to the Anterior Commissure-Posterior Commissure (AC-PC) line with TR = 300 ms, TE = 2.5 ms, bandwidth = 300 Hz/pixel, flip angle = 60°, field of view = 220 × 220 mm, matrix = 256 × 256, 32 slices with slice thickness = 4 mm and no gap. A single high-resolution T1-weighted gradient-echo scan was obtained. One hundred and seventy-six slices parallel to the AC-PC line covering the whole brain were acquired with TR = 2530 ms, TE = 3.66 ms, bandwidth = 181 Hz/pixel, flip angle = 7°, field of view = 256 × 256 mm, matrix = 256 × 256, 1 mm^3^ isotropic voxels. Functional blood oxygenation level dependent (BOLD) signals were then acquired with a single-shot gradient-echo echo-planar imaging (EPI) sequence. Thirty-two axial slices parallel to the AC-PC line covering the whole brain were acquired with TR = 2000 ms, TE = 25 ms, bandwidth = 2004 Hz/pixel, flip angle = 85°, field of view = 220 × 220 mm, matrix = 64 × 64, 32 slices with slice thickness = 4 mm and no gap. There were 300 images in each session for a total of four sessions.

Data were analyzed with Statistical Parametric Mapping (SPM12, Wellcome Department of Imaging Neuroscience, University College London, UK). In the pre-processing of BOLD data, images of each participant were realigned (motion-corrected) and corrected for slice timing. A mean functional image volume was constructed for each participant for each run from the realigned image volumes. These mean images were co-registered with the high resolution structural image and then segmented for normalization to an Montreal Neurological Institute (MNI) EPI template with affine registration followed by nonlinear transformation (Friston et al., 1994; Ashburner and Friston, 1999). Finally, images were smoothed with a Gaussian kernel of 8 mm at Full Width at Half Maximum. Images from the first five TRs at the beginning of each trial were discarded to enable the signal to achieve steady-state equilibrium between radio frequency pulsing and relaxation.

### General Linear Models of Imaging Data

Four trial outcomes—go success (GS), go error (GE), SS and stop error (SE)—were distinguished for general linear models (GLMs) of imaging data. In the first GLM, the F model, we modeled BOLD signals by convolving trial onsets with a canonical hemodynamic response function (HRF) and the temporal derivative of the canonical HRF (Friston et al., 1994). Realignment parameters in all six dimensions were entered in the model. We included the following variables as parametric modulators (PMs) in two separate F models: P(Stop) of GS trials, FP of GS trials, SSD of SS trials, P(Stop) of SS trials, FP of SS trials, SSD of SE trials, P(Stop) of SE trials, FP of SE trials. Inclusion of these variables as PM improves model fit (Büchel et al., 1996, 1998; Cohen, 1997; Hu et al., 2015b). In the F1 model P(Stop) preceded FP and in the F2 model FP preceded P(Stop) in the order of PM’s. Because SPM orthogonalizes subsequent PM with respect to antecedent PM, F1 and F2 model each allowed us to identify independent regional activities of motor preparation and stop signal anticipation. That is, FP activities were identified from the F1 model with P(stop) activities accounted for, and P(Stop) activities were identified from the F2 model with FP activities accounted for. A contrast of “+1” on the PM FP and P(Stop) each identified activities of motor preparation and stop signal anticipation from the F1 and F2 model, respectively. In actuality, as none of the 116 subjects showed a significant correlation between P(Stop) and FP (all *p*’s > 0.05), the order of these PM’s did not influence the results. Serial autocorrelation of the time series was corrected by a first degree autoregressive or AR(1) model (Friston et al., 2000; Della-Maggiore et al., 2002). The data were high-pass filtered (1/128 Hz cutoff) to remove low-frequency signal drifts.

In the second GLM, the G model, we modeled the BOLD signals by convolving go signal onsets of each trial with a canonical HRF and its temporal derivative. The goal is to identify regional activations related to RT while controlling for absolute or unsigned stimulus prediction error (UPE): |stimulus—P(Stop)|, where stimulus is 1 for a stop and 0 for a go trial (Ide et al., 2013). Thus, we included the following variables as PMs: |0–P(Stop)| or P(Stop) of GS trials, RT of GS trials, SSD of SS trials, |1–P(Stop) | of SS trials, SSD of SE trials, |1–P(Stop)| of SE trials, and RT of SE trials, in that order. A contrast of “+1” and “–1” on go trial RT each identified activities related to goRT slowing and goRT speeding.

In group level analyses, we conducted a two-sample *t*-test to compare SEQ and nSEQ for each of the four parametric contrasts: P(Stop), FP, goRT slowing and goRT speeding. For reach of these contrasts we also performed a one-sample *t* test to examine whole-brain activations across all participants. To control for type I errors, we evaluated all imaging results at *p* < 0.05, corrected for family wise error (FWE) of multiple comparisons on the basis of Gaussian random field theory, as implemented in SPM.

### Granger Causality Analysis (GCA)

As stop signal anticipation and motor preparation take place prior to motor response, we posited that neural activities associated with stop signal anticipation and motor preparation Granger causes goRT related activities. It is also likely that activities between stop signal anticipation and motor preparation as well as activities between goRT slowing and speeding may be causally related. To test these hypotheses, we employed a multivariate Granger causality analysis (GCA) to examine the direction of influence between the activities of ROIs as identified from each contrast (Granger, 1969; Stilla et al., 2007; Deshpande et al., 2008, 2009; Duann et al., 2009; Ide and Li, 2011).

The multivariate GCA was performed for individual participants. For each subject and each ROI, a summary time series was computed by averaging across voxels inside the ROI. These average time series were concatenated across sessions, after detrending and normalization (Ding et al., 2000). The pre-processed time series were used for multivariate GCA modeling. For model selection, we used Akaike Information Criterion (AIC), which imposes a complexity penalty on the number of parameters and avoids over-fitting of the data (Akaike, 1974). Multivariate GCA required that each ROI time series was covariance stationary, which we confirmed with the Augmented Dickey Fuller (ADF) test (Hamilton, 1994). The ADF test verified that there was no unit root in the modeled time series. The residuals were used to compute the Granger causality measures (*F* values) of each possible connection between ROIs. Connectivity strength was quantified by using the variance of the residual other than the sum of square of the variable (Geweke, 1982; Goebel et al., 2003), which we referred to as the *F*_Geweke_. As multivariate GCA often involves highly interdependent residuals (Deshpande et al., 2009), we used permutation resampling (Hesterberg et al., 2005; Seth, 2010) to obtain an empirical null distribution of *no causality*, as suggested by Roebroeck et al. (2005), in order to estimate the *F*_critical_, and assess the statistical significance of Granger causality. With resampling, we produced surrogate data by randomly generating time series with the same mean, variance, autocorrelation function, and spectrum of the original data (Theiler et al., 1992), as implemented in previous EEG (Kamiński et al., 2001; Kuś et al., 2004) and fMRI (Deshpande et al., 2009) studies. Following previous group analysis procedures (Sato et al., 2009; Ide and Li, 2011), we assessed the statistical significance by computing mean *F*_Geweke_ of the group for comparison with *F*_critical_, as estimated from permutation resampling (Seth, 2010). Multiple comparisons were corrected for false discovery rate (FDR; Genovese et al., 2002).

## Results

### Behavioral Performance

**Figure 1:**
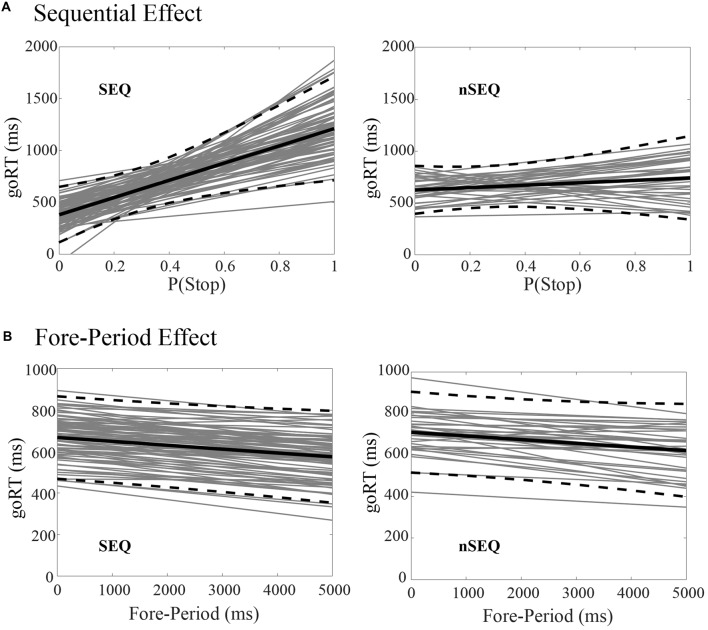
Sequential effect and fore-period (FP) effect. **(A)** Correlation between P(Stop) and go trial reaction time (goRT) across all go success (GS) trials in SEQ and nSEQ. **(B)** Correlation between FP and goRT across all GS trials in SEQ and nSEQ. Gray lines are the fitted regressions for individual participants; black solid and dashed lines are the mean and 95% confidence interval of the regressions.

Of the 116 subjects, 81 showed a significant sequential effect (*p* < 0.05; the SEQ group) and 35 did not show a significant sequential effect (*p* > 0.05; the nSEQ group). Figure 1A shows the results of linear regression between P(Stop) and goRT for individual subjects. By definition, subjects of the SEQ but not nSEQ group showed a significant sequential effect. Figure 1B shows that both SEQ and nSEQ demonstrated a significant FP effect—a negative correlation between goRT and FP—and the magnitude of FP effect was indistinguishable between the two groups. The SEQ and nSEQ did not differ in go response or SS rates. The SEQ group showed significantly shorter mean goRT and SSRT than the nSEQ group. Table 1 summarizes the performance measures from the SST.

**Table 1:** Stop signal task (SST) performance.

	SEQ (*n* = 81)	nSEQ (*n* = 35)	*T*-Value	*P*-Value
GR (%)	98.6 ± 2.7	98.2 ± 2.3	*t* = 0.6758	0.5006
SS (%)	51.3 ± 2.9	51.7 ± 3.3	*t* = −0.5697	0.5700
Median goRT (ms)	616 ± 123	675 ± 132	*t* = −2.3209	0.0221*
SSRT (ms)	207 ± 36	241 ± 48	*t* = −4.2106	0.00005*
SERT (ms)	537 ± 111	584 ± 113	*t* = −2.0739	0.0403*
FP effect (Pearson *r*)	−0.16 ± 0.10	−0.16 ± 0.13	*t* = 0.1165	0.9074

Note: SEQ/nSEQ, participants who demonstrate/do not demonstrate a significant sequential effect; GR, go response; SS, stop success; goRT, go trial reaction time; SSRT, stop signal reaction time; SERT, stop error reaction time; FP effect, linear correlation of goRT and fore-period; *p < 0.05, independent sample *t* test.

### Regional Activations to P(Stop), FP and go Trial RT

**Figure 2:**
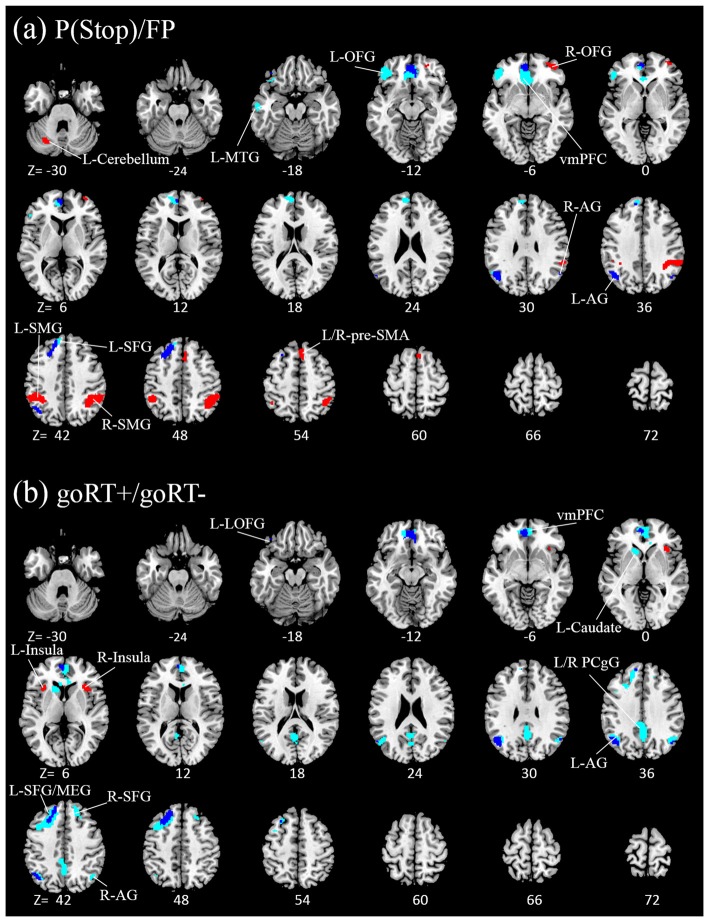
Regional activations to **(A)** stop signal anticipation (red) and motor preparation (blue) and to **(B)** go trial RT slowing (red) and speeding (blue). Clusters overlapped for motor preparation and goRT speeding. With exclusive masking, voxels distinct to motor preparation and goRT speeding are highlighted in light blue. Clusters are overlaid on a structural template in axial sections (from *z* = −30 to 72). *P* < 0.05, family wise error (FWE) corrected.

**Figure 3:**
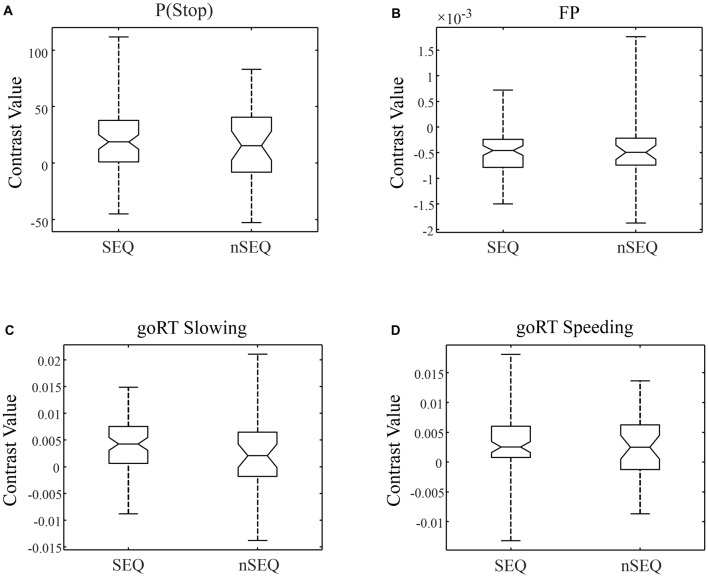
Box plots of beta weights, SEQ vs. nSEQ. **(A)** Stop signal anticipation: P(Stop); **(B)** motor preparation: FP; **(C)** go trial RT slowing; **(D)** go trial RT speeding. SEQ and nSEQ were indistinguishable for all contrasts (*p*’s > 0.05).

We compared activations to P(Stop), FP, goRT slowing and goRT speeding between SEQ and nSEQ with two-sample *t*-tests. The results showed no significant differences even when examined at a threshold of voxel *p* < 0.01, uncorrected. At *p* < 0.05, corrected for FWE of multiple comparisons, one-sample *t*-test of the entire cohort (SEQ and nSEQ combined) showed that anticipation of the stop signal engaged activations of the anterior pre-SMA, bilateral but predominantly right supramarginal gyrus (SMG), right orbitofrontal gyrus (OFG) and left cerebellum. Motor preparation was associated with activations in ventromedial prefrontal cortex (vmPFC), left OFG, left superior frontal gyrus (SFG), bilateral but predominantly left angular gyrus (AG) and left middle temporal gyrus (MTG; Table 2; Figure 2A). Go trial RT slowing was associated with activation of bilateral AI, and go trial RT speeding was associated with activation of the vmPFC, posterior cingulate cortex (PCgC), bilateral but predominantly left AG, left SFG and middle frontal gyrus (MFG), left caudate head, and left lateral OFG (Table 2; Figure 2B). Because there was spatial overlap between the clusters responding to motor preparation and goRT speeding, we constructed an additional set of ROIs for each of these two contrasts by removing overlapping voxels (Figure 2). All subsequent analyses on motor preparation and RT speeding activities were performed on ROIs following exclusive masking. Figure 3 shows the beta weight for all ROIs combined of each contrast separately for SEQ and nSEQ. It is clear that the two groups did not differ in regional response to P(Stop), FP, goRT slowing or goRT speeding. Thus, SEQ an nSEQ overall did not demonstrate differences in regional activations to conflict anticipation, motor preparation or response time adjustment.

**Table 2:** Regional activations associated with stop signal anticipation, motor preparation, goRT slowing and goRT speeding.

Contrast	Region	Cluster size (voxels)	Voxel *P*-value	Peak voxel *Z*-value	MNI coordinate (mm)		
					*X*	*Y*	*Z*
Stop signal anticipation	R OFG	69	0.000	6.18	30	53	−8
	R SMG	303	0.000	6.01	42	−52	52
	L cerebellum	26	0.002	5.54	−27	−67	−32
	L SMG	124	0.000	5.31	−45	−43	43
	R pre-SMA	56	0.000	5.20	6	26	58
Fore-period motor preparation	L OFG	151	0.000	7.55	−48	35	−11
	L AG	102	0.000	7.52	−45	−70	34
	L SFG/vmPFC	490	0.000	6.53	3	38	−14
	L MTG	17	0.003	5.57	−60	−13	−20
	R AG	16	0.003	5.40	51	−64	34
goRT slowing	R insula	55	0.000	5.89	36	20	4
	L insula	21	0.002	5.55	−33	20	7
goRT speeding	R AG	65	0.000	Inf	48	−64	40
	L AG	162	0.000	Inf	−45	−64	40
	L SFG/MFG	316	0.000	Inf	−15	38	40
	L caudate head	52	0.000	7.74	−15	17	4
	L/R PCgG	264	0.000	7.52	−3	−40	37
	vmPFC	270	0.000	7.47	0	56	1
	L lateral OFG	17	0.000	7.26	−36	35	−14
	R SFG	48	0.000	7.17	18	41	40

*Note: L, left; R, right. OFG, orbitofrontal gyrus; SMG, supramarginal gyrus; SMA, supplementary motor area; AG, angular gyrus; SFG, superior frontal gyrus; vmPFC, ventromedial prefrontal cortex; MTG, middle temporal gyrus; MFG, middle frontal gyrus; PCgG, posterior cingulate gyrus*.

### Granger Causal Relationship Between Activations to P(Stop), FP, goRT Slowing and goRT Speeding

**Figure 4:**
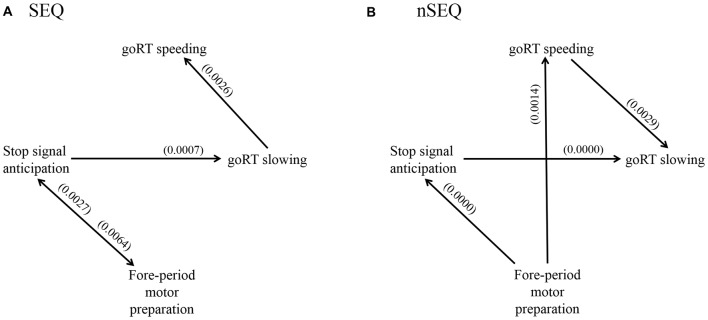
Geweke test results of Granger causality analysis (GCA) of the time series of the stop signal anticipation, FP motor preparation, goRT speeding and goRT slowing in **(A)** SEQ and **(B)** nSEQ. P(Stop) and FP activations shared reciprocal influence in SEQ but FP activities Granger caused P(Stop) activities unidirectionally in nSEQ. Further, FP activities Granger caused goRT speeding activities in nSEQ but not SEQ. Numbers in parenthesis indicate the *p* value for that connection, corrected for false discovery rate (FDR).

We used GCA to examine the directional connectivity between brain regions responding specifically to stop signal anticipation, motor preparation and RT modulation. We combined the clusters as a single ROI for each of the four constructs and evaluated the significance of each connection, i.e., Geweke test (*F*_Geweke_ > *F*_critical_), for SEQ (*n* = 81) and nSEQ (*n* = 35). In SEQ, the results of the Geweke test showed significant connectivity for: P(Stop) ↔ FP; P(Stop) → goRT slowing; and goRT slowing → goRT speeding (Figure 4A). In nSEQ the results of the Geweke test showed significant connectivity for: FP → P(Stop); P(Stop) → goRT slowing; FP → goRT speeding; and goRT speeding → goRT slowing (Figure 4B).

We also considered the number of participants with a significant connectivity in each group and employed one-tailed Fisher’s exact test to directly compare SEQ and nSEQ for each connectivity. The results showed a significant difference for FP → goRT speeding (30/81 of SEQ vs. 20/35 of nSEQ, *p* < 0.036). The *p* value for P(Stop) → FP was 0.145 (38/81 of SEQ vs. 12/35 of nSEQ). All other *p* values were >0.246.

## Discussion

There are several main findings. First, individuals who demonstrated a sequential effect (SEQ) and those who did not (nSEQ) were indistinguishable in the magnitude of FP effect. Both showed a significant negative correlation between FP and go trial RT and the magnitude of correlation did not differ between the two groups. Second, the two groups did not differ in regional activations to P(Stop), FP, goRT slowing or goRT speeding, even when the results of two-sample *t* tests were examined at a very liberal threshold, suggesting that P(Stop) is represented in both SEQ and nSEQ, with shared regional activities for proactive control. Third, Granger causality analyses identified differences in the interactions between ROIs in response to P(Stop), FP, goRT slowing and goRT speeding. Specifically, both SEQ and nSEQ demonstrated a Granger causal influence of P(Stop) activities on goRT slowing activities. However, in contrast to SEQ where there are bidirectional influences between P(Stop) and FP activities, the Granger causality is significant only from FP to P(Stop) activities—suggesting a predominance of motor preparation—in the nSEQ. Further, nSEQ but not SEQ demonstrated a significant directional influence of FP on goRT speeding activities, and SEQ and nSEQ showed opposite directions of influence between activities of goRT slowing and speeding: slowing activities Granger caused speeding activities in SEQ and* vice versa* in nSEQ. Together, these findings suggest that although both SEQ and nSEQ demonstrate a significant and indistinguishable FP effect and similar regional activations to conflict anticipation and motor preparation, motor preparation in nSEQ influences conflict anticipation and goRT speeding to such an extent, that it significantly diminishes the strategic adjustment of response time to fluctuating P(stop) and thus the sequential effect. Although GCA has its limitation in addressing causal relationship between regional time series (Friston, 2009) and cannot elucidate causal interaction in an event-related manner, it has been widely used to support directional functional connectivity between regional activities (Roebroeck et al., 2005; Seth, 2010). Here, along with our earlier work (Duann et al., 2009; Hu et al., 2015a), we were able to employ GCA to highlight the directional interactions between regional processes of motor preparation and proactive control.

To our knowledge, these are the first set of findings to characterize how motor preparation interacts with conflict anticipation to determine motor response in the SST. Conflict anticipation involves predominantly right-hemispheric brain regions, including the pre-SMA and SMG (Hu et al., 2016). In contrast, motor preparation engages predominantly left-hemispheric areas including the caudate head, AG and SFG as well as vmPFC and PCgC, core areas of the default model network (DMN). The pre-SMA is widely implicated in self control (Jaffard et al., 2008; Rushworth, 2008; Sharp et al., 2010; Cieslik et al., 2015; Hampshire and Sharp, 2015), and unlike the more posterior medial frontal structures that project to the lentiform nucleus, the pre-SMA projects to the caudate head (Zhang et al., 2012), in support of a hierarchical structure where anterior and posterior medial prefrontal regions each respond to task set and response control (Korb et al., 2017). As pre-SMA responds to conflict anticipation and the left caudate nucleus responds to motor preparation, it is likely that the right-hemispheric pre-SMA interacts with the left caudate head via its excitatory inputs to the right caudate head, which in turn inhibits the left caudate head through trans-hemispheric processes or subcortical mechanisms involving the pallidum (Watanabe et al., 2015). In another study, anodal transcranial direct current stimulation of the pre-SMA improved SSRT along with changes in pre-SMA connectivity with the vmPFC during stop trials (Yu et al., 2015). In contrast, “lesioning” by repetitive transcranial magnetic stimulation of the left IPC, which responds to motor preparation, reduced risk taking—a behavioral analog of speeded response—in a gambling task (Coutlee et al., 2016). Together, these findings support an interaction between the conflict anticipation and motor preparation circuits.

The left lateral orbitofrontal cortex (OFC) responds to motor preparation. The OFC comprises multiple subregions each implicated in distinct roles to support motivated behavior (Dixon et al., 2017). The left OFC increased activation to reward decision making in association with a behavioral approach personality trait (Yamamoto et al., 2017) and to loss trials when individuals know they are more likely to lose than not (Dong et al., 2013). Anatomically the lateral OFC is heavily connected with the somatomotor and premotor structures as well as the amygdala (Cavada et al., 2000). These studies suggest that left OFC activation may reflect the affective component of motor urgency accompanying the premotor processes. The left SFG also responds to motor preparation and goRT speeding. Patients with left SFG lesions exhibited a working memory deficit and the impairment increased with task complexity, most markedly for the spatial domain (du Boisgueheneuc et al., 2006). In a semantic task, the left SFG increased activation to a longer “dwell” time before response (Scott et al., 2003). These findings support a role of the left SFG in memory related processing during the FP when participants anticipate ending the wait, and contrast with the role of the right-hemispheric SFG in restraining a motor response (Dambacher et al., 2014; Hu et al., 2016). Also of note is the activation of the vmPFC and PCgC—core structures of the DMN—during response speeding. The DMN is commonly “deactivated” when participants are engaged in external task challenges. Thus, responding “as usual” to the frequent go signal may represent a default behavioral state and the DMN deactivates when there is an impending need to stop.

The current fMRI study is also the first to characterize the neural correlates of FP effects in the SST. A recent work employed a cued reaction time task to examine the neural processes of temporal expectation, where the target appeared after one of four intervals (FPs) that was either predictable (temporal condition) or variable (neutral condition; Coull et al., 2016). As expected, RTs were faster in temporal vs. neutral condition, indicating the behavioral benefit of temporal predictability. RTs were also faster as a function of FP in the neutral, but not temporal, condition. The IPC, in the area of the SMG, showed greater activation in the temporal vs. neutral condition and along with increasing FP in the neutral but not temporal condition. This finding supported the role of the IPC in temporal expectation and response control. However, it appears at odds with the current findings as we showed SMG response to conflict anticipation rather than motor preparation. Importantly, participants were required solely to execute a speedy response (RT ~300 ms vs. ~650 ms here) in the temporal expectation task. It is possible that, involved in flexible sensorimotor mapping (Randerath et al., 2017), the SMG is versatile in response to task requirements and engaged to execute either a speedy or constrained action as the task requires. It is also notable that the SMG and AG seem to be differentially involved in response slowing and speeding. The SMG and AG exhibit distinct functional connectivity with the SMG more heavily connected with the ventrolateral prefrontal cortex—a region involved in post-error slowing in the SST (Ide and Li, 2011)—and the AG more connected with the DMN (Daselaar et al., 2013; Zhang and Li, 2014). More work is needed to investigate whether the two inferior parietal structures are involved in opposing psychological processes beyond motor response control and whether these functions may be lateralized hemispherically.

An intriguing finding concerns the SSRT, which is significantly shorter in the SEQ, suggesting more efficient response inhibition, than the nSEQ. This appears to make sense on the surface, as motor preparation may not only compromise proactive control but also impede the stopping process and prolong SSRT (Castro-Meneses et al., 2015). In support, the pre-SMA not only responds to conflict anticipation but also represents a critical node in the cortical-subcortical circuits to support response inhibition (Duann et al., 2009). On the other hand, as described earlier, tracking the stop trial, in contrast to assuming a constant frequency of stop signal, establishes the sequential effect but should not bear on other aspects of stop signal performance. The relationship of sequential effect with the SSRT thus does not seem amenable to simple mechanistic explanation, and motivational factors may facilitate response inhibition in SEQ.

To summarize, we characterized how motor preparation interact with conflict anticipation to determine motor response in the SST. Conflict anticipation involves a network of predominantly right-hemispheric regions, including the pre-SMA and SMG, whereas motor preparation engages predominantly left-hemispheric areas including the caudate head, AG and SFG as well as core structures of the default mode network. These regional activities do not distinguish individuals who demonstrate a sequential effect and those who do not. That is, conflict anticipation or P(Stop) is represented neutrally in nSEQ, similar to SEQ. However, nSEQ do not strategically optimize their decision policy given the prior information encapsulated in P(Stop). A multitude of factors, including motivation, could explain why nSEQ do not adjust response time according to P(Stop). Here, we showed that functional connectivity of motor preparation predominates and may disrupt proactive control in the latter individuals. The findings suggest the complexity of SST performance and provide evidence to support an interaction between the go and stop processes (Boucher et al., 2007; Yu and Cohen, 2008).

## Author Contributions

AY and CRL designed the experiment. WW, SH, JSI, SZhornitsky and SZhang carried out the study. All authors contributed to literature review and writing of the manuscript.

## Conflict of Interest Statement

The authors declare that the research was conducted in the absence of any commercial or financial relationships that could be construed as a potential conflict of interest.
